# Unqualified Assistants for Infirmary Staffs

**Published:** 1914-10-03

**Authors:** 


### UNQUALIFIED ASSISTANTS FOR INFIRMARY STAFFS.

The proposal made by Dr. Baly, medical superintendent of the Lambeth Infirmary, referred to in our Notes last week, to staff the infirmary partly with unqualified men in their last year of training, is one which will not commend itself, we think, either to the Local Government Board or to the medical profession. We can sympathise with Dr. Baly in the difficult position in which he finds himself with three of his six medical officers withdrawn from him by the war. His suggestion does not appear, however, to solve the difficulty. In the first place we doubt if it will be so easy to find suitable unqualified men in their last year willing to take these posts for an honorarium of 15 guineas a quarter, with residential allowances. Why should they do so? Obviously at present the object of every unqualified man is to get qualified as soon as the regulations will permit. Residence at a Poor-Law infirmary, extremely valuable as is the experience to be gained there, cannot compare with the systematic clinical instruction at a teaching hospital for the purposes of a student preparing for his examinations. But even if suitable candidates are forthcoming, the amount of assistance they can give is severely limited by the stringent, and rightly stringent, regulations of the General Medical Council. That the Council will not permit any relaxation of these regulations is apparent from the warning letter recently published in the medical papers. The work of the unqualified assistant should be confined to the carrying out of clinical examinations under the direct supervision of the qualified medical officers, and diagnosis and treatment should be retained in the hands of the latter. In all Poor-Law infirmaries the medical staff is barely sufficient to carry out these essential duties efficiently. Unqualified clinical assistants would be of value as adjuncts to the staff by carrying out pathological examinations and detailed investigations for which the existing staffs have little or no time; but they cannot replace them.

The whole difficulty is really a financial one. The salary attached to the post of junior assistant medical officer at the Lambeth Infirmary is £150 per annum. For similar posts other public bodies are offering considerably higher salaries. The Metropolitan Asylums Board, for instance, are now paying the junior medical officers at their hospitals £250. For years past the Poor-Law medical service has been underpaid in all its grades. Long before the war there was difficulty in obtaining suitable candidates at the salaries offered. Of recent years the difficulty has increased owing to the large number of new posts open to newly qualified men and to the higher salaries paid to assistants in private practice. The war has come as a climax which has effectively swept away the small number of men formerly willing to accept these poorly paid posts. It is not only that the junior posts are underpaid. The number of medical superintendentships is small, promotion to them is far too much a matter of chance. Even so, if these posts were highly paid the possibility of obtaining one would attract men to the service. Unfortunately the reverse is the case. Boards of Guardians in the past have often succeeded in filling these posts by offering them to the assistant medical superintendent at an absurdly inadequate salary. The Local Government Board, which might be expected to take wider views and to realise the importance of maintaining the attractions of the service, has sanctioned these bargains and must take its share of responsibility for the deadlock which has now arisen.

The solution is to be found not in reducing the efficiency of the service by accepting unqualified candidates, but by increasing its attractions, as the Metropolitan Asylums Board has realised. When the prospects in the Poor-Law medical service have been brought into line with the improved conditions in the other public medical services and in private practice, then and then only will the present dearth of candidates disappear. Even if, as the war goes on, the demands of the Army and Navy should lead to an actual shortage of medical men—and that time has not yet arrived—there is another and better way of meeting the emergency than Dr. Baly's proposal. In the case of Lambeth, where some of the districts are worked by the infirmary staff, the medical work in the districts could be given temporarily to private practitioners. Even in the infirmaries private practitioners in the neighbourhood could be engaged to attend for a few hours daily, and so give real and efficient help to a reduced staff. This is the solution which, a correspondent stated in our issue of September 19, has been adopted at the *Dreadnought* Hospital to meet losses directly due to the war, and is one which presents no insuperable difficulties in the case of a Poor-Law infirmary.

